# Development and Application of an Additively Manufactured Calcium Chloride Nebulizer for Alginate 3D-Bioprinting Purposes

**DOI:** 10.3390/jfb9040063

**Published:** 2018-11-09

**Authors:** Lukas Raddatz, Antonina Lavrentieva, Iliyana Pepelanova, Janina Bahnemann, Dominik Geier, Thomas Becker, Thomas Scheper, Sascha Beutel

**Affiliations:** 1 Institut für Technische Chemie, Leibniz Universität Hannover, Callinstraße 5, 30167 Hannover, Germany; raddatz@iftc.uni-hannover.de (L.R.); lavrentieva@iftc.uni-hannover.de (A.L.); pepelanova@iftc.uni-hannover.de (I.P.); jbahnemann@iftc.uni-hannover.de (J.B.); scheper@iftc.uni-hannover.de (T.S.); 2 Lehrstuhl für Brau- und Getränketechnologie, Technische Universität München, Weihenstephaner Steig 20, 80354 Freising, Germany; dominik.geier@tum.de (D.G.); tb@tum.de (T.B.)

**Keywords:** 3D printing, bioprinting, alginate, bioink, cell culture technology, rapid tooling, hydrogels, customizable labware

## Abstract

Three-dimensional (3D)-bioprinting enables scientists to mimic in vivo micro-environments and to perform in vitro cell experiments under more physiological conditions than is possible with conventional two-dimensional (2D) cell culture. Cell-laden biomaterials (bioinks) are precisely processed to bioengineer tissue three-dimensionally. One primarily used matrix material is sodium alginate. This natural biopolymer provides both fine mechanical properties when gelated and high biocompatibility. Commonly, alginate is 3D bioprinted using extrusion based devices. The gelation reaction is hereby induced by a CaCl_2_ solution in the building chamber after material extrusion. This established technique has two main disadvantages: (1) CaCl_2_ can have toxic effects on the cell-laden hydrogels by oxygen diffusion limitation and (2) good printing resolution in the CaCl_2_ solution is hard to achieve, since the solution needs to be removed afterwards and substituted by cell culture media. Here, we show an innovative approach of alginate bioprinting based on a CaCl_2_ nebulizer. The device provides CaCl_2_ mist to the building platform inducing the gelation. The necessary amount of CaCl_2_ could be decreased as compared to previous gelation strategies and limitation of oxygen transfer during bioprinting can be reduced. The device was manufactured using the MJP-3D printing technique. Subsequently, its digital blueprint (CAD file) can be modified and additive manufactured easily and mounted in various extrusion bioprinters. With our approach, a concept for a more gentle 3D Bioprinting method could be shown. We demonstrated that the concept of an ultrasound-based nebulizer for CaCl_2_ mist generation can be used for 3D bioprinting and that the mist-induced polymerization of alginate hydrogels of different concentrations is feasible. Furthermore, different cell-laden alginate concentrations could be used: Cell spheroids (mesenchymal stem cells) and single cells (mouse fibroblasts) were successfully 3D printed yielding viable cells and stable hydrogels after 24 h cultivation. We suggest our work to show a different and novel approach on alginate bioprinting, which could be useful in generating cell-laden hydrogel constructs for e.g., drug screening or (soft) tissue engineering applications.

## 1. Introduction

One of the most important achievements of mankind is the invention of the printing by Johannes Gutenberg in 1450 AD. With this new method, the typesetting and printing of books was made easier, faster, and less expensive than ever before [1]. Book prices dropped and common people obtained access to literature, which is often referred to as the trigger for “democratization of knowledge” and paved the way for the Reformation and the European Enlightenment [2]. Today, about 570 years later, another disruptive technology revolutionizes our society: three-dimensional (3D) printing. Compared to conventional production strategies this technology enables the fabrication of complex structures in very short periods of time and at lower costs. Because of these advantages, it could, analogously to Gutenberg’s invention, initiate the “democratization of manufacturing”. Since its invention in the 1980s and after significant impact on the production industry, 3D printing currently finds application in biotechnological and chemical laboratories [3,4,5,6,7]. The current research generally confirms that it enables scientists to generate custom tissue constructs for cell screening. In the past years, the disadvantages of conventional two-dimensional (2D) cell culture for pharmaceutical research and industry have become more apparent, as accurate in vitro assays turn out to be of poor predictive value [8] caused by reduced cell-cell contacts in 2D cell cultivation. The extra cellular matrix (ECM) generation is limited significantly, resulting in completely different cell behavior compared to in vivo 3D tissues [9]. Bioprinting combines 3D printing technology with cell culture technology and it enables researchers to additively manufacture custom structures containing living cells and to imitate tissues [10]. The use of hydrogels as the biomaterials in 3D bioprinting has been described intensively and the most frequently used methods involve: laser-induced forward transfer (LIFT), inkjet printing (IP), and extrusion or dispensing based (EB) [11,12,13]. Despite its comparable low resolution of about 200–500 µm in the x/y plane, EB’s fabrication speed is significantly higher compared to other methods and is often described as the most promising among bioprinting techniques [11,14,15]. One main aspect of 3D bioprinting and bioink compositions are the physical properties of the tissue that shall be imitated—the spectrum reaches from hard cartilage and bone tissue [16] to super soft tissue. The latter was realized using cryogenic 3D printing methods and poly(vinyl) based bioinks. With this technique, constructs can be manufactured, which are of comparable softness as brain and lung tissue [17]. Besides hydrogels as bioinks, silicone compositions are frequently used, as well. Due to their biocompatibility, oxygen permeability, and porous structure, they are suitable for several applications in 3D bioprinting. However, despite its use for the mimicry of mechanical structures, its applicability is limited and e.g., not suitable for drug release or direct cell to cell studies [18]. Recent innovations led to the fabrication of a heart valve using an alginate based bioink [19], but it is still far away from generating transplantable tissues. On the other hand, functional human tissue for drug testing [20], collagen-derived natural leather, and food [21] are already purchasable—to list a few examples. The most frequently used bioprinting materials are hydrogels, which are water retaining insoluble polymers consisting of e.g., proteins and carbohydrates. The highly biocompatible substances, allow cell remodeling, attachment, and spreading. Ideally, the gel undergoes a quick and controlled sol-gel transition, through a change in temperature or pH. Some hydrogels, however, have to be modified chemically to allow fast curing during or after 3D bioprinting. A high variety of materials are used for 3D bioprinting, among which gelatin methacrylate, collagen/gelatin, fibrinogen, agarose, methacrylated hyaluronic acid, and alginate play an important role. Also, compositions are used in order to improve cell-hydrogel interactions or printability [22]. Sodium alginate (Na/Alg) based bioinks have already been extensively studied and numerous publications demonstrate their high potential and wide range of applicability. Na/Alg is a salt derived from the alginic acid and forms a water insoluble hydrogel by the addition of divalent ions, such as Ca^2+^. It is biodegradable, non-toxic, non-immunogenic, and composed of linear polysaccharides (guluronic and mannuronic acids) [23]. This biopolymer gets utilized both as stand-alone material and as an additive for bioink compositions.

Several groups are using cell-laden alginate hydrogels containing primary hepatozytes, HUVECs, or human chondrocytes in order to manufacture respective tissue at some point [24,25,26,27,28]. Furthermore, microphysiological systems and vascular graft structures were successfully bioprinted and described [25,29]. Sodium alginate is polymerized by ionic crosslinks with CaCl_2_. Normally, in order to convert the liquid alginate to a gel, the cell-laden bioink is printed directly into a CaCl_2_ solution. Often, this extensive and complex-to-handle procedure leads to inadequate results. The post-processing, including construct transfer to a cultivation vessel, can lead to its destruction and can influence the cell viability. Moreover, oxygen transfer to the cells in the biomaterial is inhibited when 3D bioprinted directly into a CaCl_2_ solution. Other researchers are facing this issue by using alginate containing composites aiming to improve construct quality and hydrogel properties. “CELLINK” is a commercially available bioink that combines nanocellulose and alginate. This hydrogel has fast crosslinking features, making it relevant for soft tissue applications producing more stable objects [10,23]. In other approaches using gelatin [30] or chitosan as accessory agent successfully improved bioprinting results [30,31]. However, the mentioned studies use alginate composite bioink. Another advance was made establishing a different curing method. The freeform reversible embedding of suspended hydrogels (FRESH) termed method utilizes a secondary hydrogel (for example gelatin) functioning as a temporary support [32]. The support matrix contains CaCl_2_ inducing the gelation immediately. It could be shown that soft hydrogel scaffolds can be manufactured using FRESH [32], although major disadvantages include the low product resolution, and again, its destruction during post processing and gelatin wash off.

We have developed a novel crosslinking approach using a custom 3D printed CaCl_2_ nebulizer. This method allows for a decrease of CaCl_2_ concentration to avoid possible toxic effects on the cells and to improve construct grip to the culture plate surface. Current protocols use either a higher CaCl_2_ concentration, a significant longer incubation time of the constructs in the curing solution, or combinations of different polymers [19,27,33]. In this study, the nebulization chamber LR06 was designed using CAD software and was printed with a multi jet printer (MJP). The LR06 provides high mist density without affecting the bioprinting process utilizing a piezo element. By applying alternating voltage to the element, the crystal starts to oscillate. This movement is transferred to the above-placed CaCl_2_ solution inducing compression waves in the liquid. At the surface of the solution, the high kinetic energy of the waves leads to the nebulization of the liquid, yielding a mist. Two theories are discussed to explain the process: (1) The capillary waves theory postulates that the nebulization takes place at the crests of instable waves at the liquid surface. According to (2), the cavitational bubble theory, a different effect is responsible for the nebulization: Caused by pressure differences between the compression waves, small gas bubbles deploy and implode, generating the mist [34,35,36,37,38]. The exact nebulization mechanism is yet not understood entirely [35]. Different concentrations of alginate were characterized in terms of viscosity and stiffness after polymerization. We successfully 3D bioprinted adipose-derived mesenchymal stem cells and murine fibroblasts (NIH-3T3-GFP) suspended in different alginate concentrations. The cells remained viable and the hydrogels stable during cultivation time. We successfully combined the nebulizer with the Allevi (Allevi Inc., Philadelphia, PA, USA) bioprinter Biobot 1. By reason of its customizable building plate-mount, the LR06 can easily be adjusted to other bioprinter models. The therefore needed CAD .stl blueprints are provided and are ready to download as supplementary material (Files S1 and S2).

## 2. Results

### 2.1. Design, Manufacturing and Functioning of the CaCl_2_ Nebulizer

**Figure 1:**
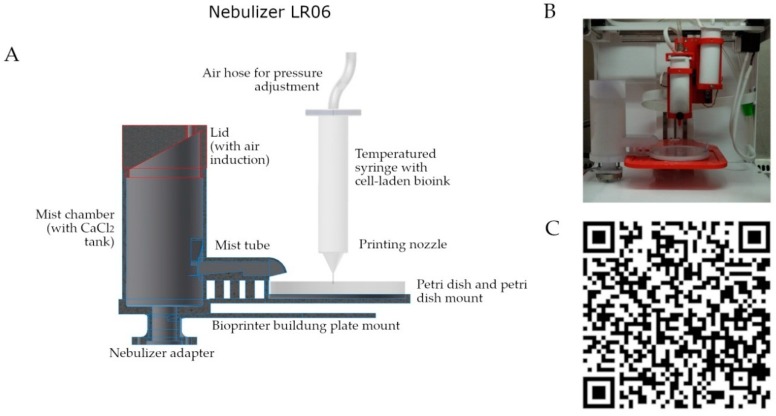
Additive manufactured CaCl_2_ Nebulizer LR06. (**A**) Schematic representation of the LR06 and extruder nozzle; (**B**) Photograph of the LR06 mounted to a bioprinter; (**C**) A demonstration video of the LR06 can be found in the supplementary section of the journal and linked as a QR-Code.

The presented 3D bioprinting system is a novel development and uses nebulized CaCl_2_ in order to induce Na/Alg polymerization reaction. The main parts of the system (lid and mist chamber with petri dish mount, displayed in grey, Figure 1) were designed using CAD software and additively manufactured with a MJP 3D printer. Using this method, high definition products can be manufactured in short periods of time and of high quality material. The printing material (epoxy resin) is further discussed in Section 2.1.1. The device was designed for the Allevi 1 bioprinter, but it can be modified in CAD and adapted for other extrusion-based bioprinters. The second part of this device is the nebulizer, mounted to the nebulizer adapter (Figure 1). For the mist generation, it uses a piezo element (piezo crystal). For the whole experimental set-up and workflow, Figure 1C provides the hyperlink to a demonstration video or see supplementary material (Video S1).

#### 2.1.1. Toxicity Evaluation of the Printing Material and Nebulization Effect

**Figure 2:**
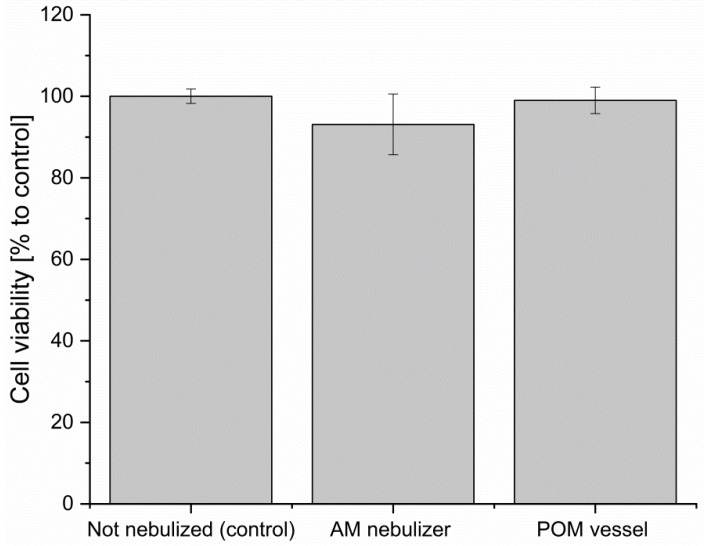
Evaluation of additive manufactured (AM) material used for the manufacture of the LR06. Cell viability of hAD-MSCs in cell culture media that was previously nebulized using: the LR06 (middle column), a vessel made of inert material (POM) (right column). As the control untreated media was used (left column). Cell viability is high (above 90%) in both experimental set-ups indicating, that no negative effect was generated during the nebulization process. Data points are means and SD for *n* = 3. Statistical analysis of the data found no significant difference of shown means (ANOVA One Way, *p* = 0.24).

The here demonstrated 3D printed nebulizer is made from a resin polymer [39] and it has to fulfill important characteristics, like chemical stability. The printing technique was already evaluated in previous studies, as were the fabricated materials for potential leaching of the cured resin when in contact with biological compounds. It could be shown that the use of this material has no negative influence on the cell viability of animal cell lines (L-929 and NIH-3T3) and cell growth of microbial cells (*E. coli*) [4]. During the ultrasound-driven nebulization process, the liquid is exposed to a high amount of kinetic energy. Material debris could reach the 3D printing platform carried by the mist. In order to evaluate the potential toxic effects on the cell culture, a cytotoxicity assay was performed. Therefore, conventional cell culture media (alpha-MEM) was nebulized both in the LR06 and a second (non-3D-printed) vessel of high performance polyoxymethylene (POM) plastic. The nebulization took place with complete reflux to the tank. This media was further used for a cultivation (mesenchymal stem cells) and a cell viability assay (CellTiterBlue^®^). With this set-up, the cell viability of the cells cultivated in (1) the native media (control), (2) the nebulized media (without risk of debris contamination), and (3) the nebulized media in the LR06 can be evaluated. The results of the cell viability assay are shown in Figure 2. The cell viability of both samples is distinctively above 90% to control, indicating no negative effect on the cells (referring to ISO-10993-5:2009) [40]. The slight decrease of 1% (POM) and 6.9% (LR06) in cell viability as compared to the control notable in Figure 2 could not be statistically validated.

### 2.2. Hydrogel Characterization

In order to be suitable as biomaterials for 3D bioprinting, hydrogels must have certain properties, as described above. The viscosity of alginate solutions (Na/Alg) and the storage modulus of crosslinked alginate (Ca/Alg) were determined for various relevant concentrations.

#### 2.2.1. Viscosity

**Figure 3:**
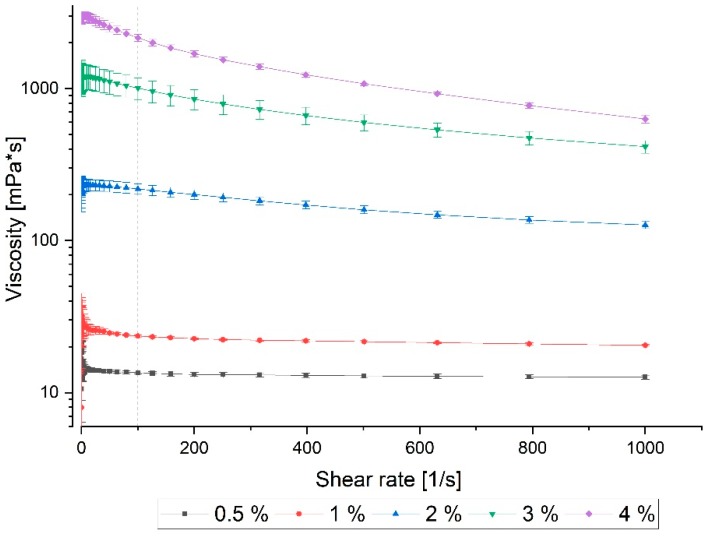
Rheological determination of the viscosity of different sodium alginate concentrations (% (*w*/*v*)). Data was obtained by rotational viscometry at 37 °C and logarithmic increase of the shear rate. 2–4% samples show a shear-thinning behavior making them suitable for 3D bioprinting. Data points are means and SD for *n* = 3.

Viscosity of five Na/Alg concentrations (0.5%, 1%, 2%, 3%, and 4% (*w*/*v*)) was determined with the help of rheometry. Figure 3 demonstrates the viscosity behavior over a temporal increase of the shear rate. Results show that an increase in alginate concentration leads to a higher material viscosity, going along with the current literature. The lowest concentrations of 0.5–1% (*w*/*v*) alginate show the lowest viscosity of 13.5 mPa s (at 100 1/s) and 23.1 mPa s (at 100 1/s). The solution with 2% (*w*/*v*) shows a nine-fold higher viscosity of 218.6 mPa s (at 100 1/s) and a further concentration increase results in even higher viscosities: 3% (*w*/*v*): 1007.7 mPa s (at 100 1/s) and 4% (*w*/*v*): 2156.6 mPa s (*w*/*v*).

Furthermore, the solutions with 2–4% alginate show a reduction of viscosity at increasing shear rate. This material behavior is typical for some polymers and it is called the shear-thinning effect [41]. Especially for extrusion 3D printing applications, a shear-thinning behavior of the bioink is desired. It leads to a better fluid flow when the material undergoes a certain force, as it is predominant in the extrusion nozzle during 3D bioprinting. Because of the pressure-induced shear stress, the viscosity decreases, which results in easier hydrogel deposition. Once the stress is removed, the viscosity increases again, leading to improved shape stability. In this case, a shear-thinning effect can be observed at alginate concentrations of 2% and higher.

#### 2.2.2. Storage Modulus

The storage modulus (G’) was determined together with G’’ in oscillation rheology. The G’ was always higher than the G’’, indicating the all tested constructs behaved like solids. Furthermore, G’ stayed at the same level throughout the measurement, indicating that polymerization was complete [41].

**Figure 4:**
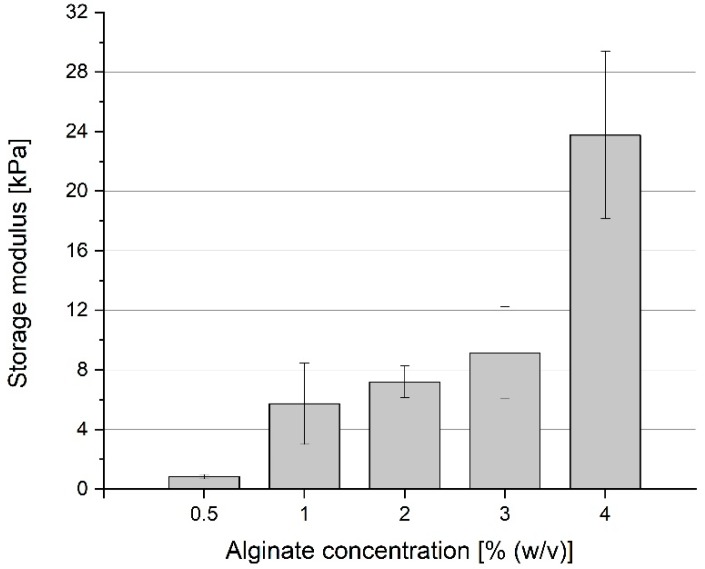
Effect of Cross-linking at five different alginate concentrations on the Storage modulus (G’). G’ was determined using model constructs (2 cm diameter, 1 mm height) by performing a time sweep in an oscillation experiment. Data points are means and SD for *n* = 3.

Figure 4 shows the storage moduli of different alginate concentrations. As expected and according to the literature, the storage moduli increase with increasing concentration. Whereas, the storage modulus of the lowest alginate concentration (0.5%) is about 834 ± 143 kPa, the highest alginate concentration of 4% = 23.767 ± 5.614 kPa. The other three samples G’ values in between this range and vary from 5.738 ± 2.729 to 9.144 ± 3.078 kPa.

### 2.3. Bioprinting

Before actual bioprinting with stem cells (hAD-MSCs) and murine fibroblasts (NIH-3T3-GFP) was conducted, the bioprinting parameters of used hydrogels were determined (Table 1).

#### 2.3.1. Bioprinting Parameters and Shape Evaluation

Table 1 shows the used bioprinting parameters for all experiments. The alginate hydrogels with the lowest concentrations (0.5% and 1% (*w*/*v*)) did not show shear-thinning properties in rheological experiments. These findings go together with poor resolution and shape fidelity in the printing results in this set-up. These concentrations were not used for further bioprinting with cells (Table 1).

**Figure 5:**
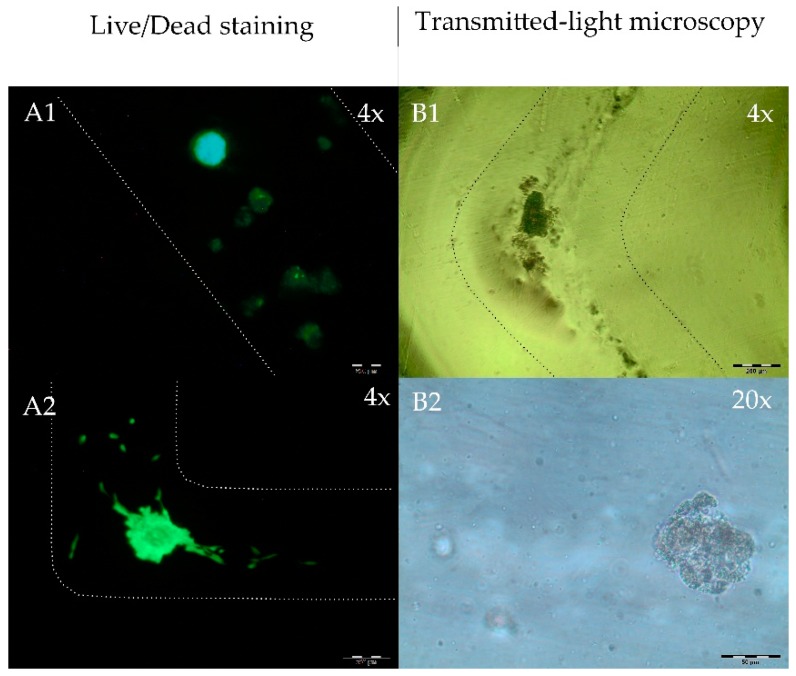
Microscopic images of human adipose tissue-derived mesenchymal stem cells (hAD-MSC) spheroids in Ca/Alg hydrogel. Images were taken right after three-dimensional (3D)-bioprint using the LR06. All images show different sections. The borders of the lanes were marked for better visibility. (**A**) Live/dead (Calcein AM/Propidium Red) images in 4× magnification (scale bar = 200 µm). In subfigures (**A1**) and (**A2**) the majority of the cells show green fluorescence and is viable. (**B**) Transmitted-light microscopy showing one corner of the 3D printed construct indicating the precision of the method ((**B1**), 4×) and a zoom on one spheroid ((**B2**), 20×, scale bar = 50 µm). Spheroid diameter is 50–200 µm.

**Figure 6:**
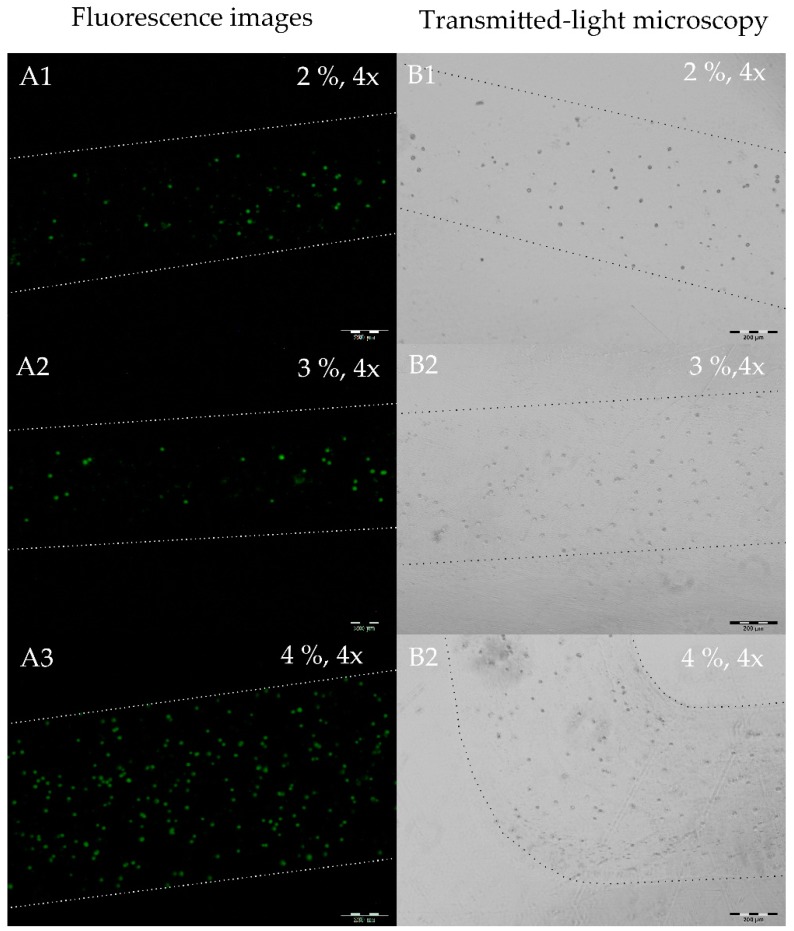
Microscopic images of NIH-3T3-GFP fibroblasts of different alginate-concentrations (2%, 3% and 4% (*v*/*w*)) in 4× magnification (scale bar = 200 µm). The images were taken after 3D-bioprint using the LR06 and 24 h cultivation. All images show different sections. The borders of the lanes were marked for better visibility. (**A**) Fluorescence microscopy images. (**A1**–**A3**) show green fibroblasts due to GFP expression. (**B**) Transmitted-light microscopy images. (**B1**–**B3**) show the transmitted-light microscopy images. (**B3**) shows a 90° angle of the 3D-bioprinted structure.

In Figure 5 and Figure 6 printing results with different alginate concentrations containing cells are shown. The printing line diameter averages at 500 µm. The adherence on the surface was strong enough to let the constructs stick to the petri dish surface during cultivation. The constructs’ theoretical side length (please see supplementary material, Figure S1) was supposed to be 9 mm with the inner lines being 5 mm long. Actual results show 11 mm sides and a 6.5 mm inner line length. Bioprinting of non-linear geometries can be critical and it is often avoided. Figure 6 (bottom right) also shows that narrow curves were manufactured without loss in shape quality or material diffluence.

#### 2.3.2. HAD-MSC Bioprinting

Bioprinting with stem cells obtained the best results using 3% Ca/Alg concentration—shown in Figure 5. Average printing line diameter is 400 µm. A critical area in terms of product quality are corners in the printed object. Figure 5 (top right, bottom left) shows that this area could be manufactured without diffluence of the bioink. The images were taken after 24 h cultivation time and show both cell types after live/dead staining. The majority of the cells are still viable and survived both the bioprinting process intself and CaCl_2_ mist-induced bioink polymerization. The majority the of cells in the live/dead image show green fluorescence and are viable.

#### 2.3.3. NIH-3T3 3D Bioprinting

In addition to the printed stem cells that are presented above, murine fibrobasts were 3D bioprinted while using the LR06. The used cell line was transduced with a green fluorencence protein. This allows for observing the cells under fluorescent light and track cell viability via GFP protein biosynthesis. The printing results for three different Ca/Alg concentrations are presented in Figure 6. The fibroblasts’ green appearance is originated in GFP expression in the hydrogel in all 3D bioprinted alginate concentrations, indicating viable cells.

## 3. Discussion and Conclusions

Recent research demonstrates the importance of Na/Alg 3D bioprinting and its many applications [19,23,24,25,26,27,28,29]. The here introduced 3D printed nebulizer (LR06) is an innovative approach of Na/Alg 3D bioprinting and it is realized by combining two additive manufacturing methods: (1) The presented nebulizer LR06 was 3D printed using MJP technology and designed for (2) extrusion based 3D bioprinting applications. We demonstrated that this set-up is suitable for manufacturing alginate constructs. This allows the assumption that its application in regulated fields with high standards, such as the biotechnological sector and tissue engineering, is possible. No negative effects on investigated cell lines that came in contact with the generated mist could be measured. The used cells showed no reduction in cell viability compared to the control. In actual application of the LR06, however, no media but CaCl_2_ gets nebulized and is washed off immediately after crosslinking, minimizing the risk of toxic effects.

Besides the development of LR06, the characterization of suitable alginate concentration for bioprinting was carried out. Viscosity and storage modulus (G’, describing the behavior of a visco-elastic body under oscillatory shear stress) of Na/Alg, and Ca/Alg in concentrations of 0.5–4% (*w*/*v*) were determined. Corresponding to existing literature, we showed that concentrations of 2% and higher have shear-thinning material properties, a relevant characteristic of a biomaterial for extrusion bioprinting [42]: The pneumatically applied pressure on the bioink improves its fluid flow behavior and it enhances the construct to hold its shape after extrusion process and before completion of the polymerization. Storage modulus (G’) of the alginate was determined for the same alginate concentrations and an increase with higher Ca/Alg concentration could be evaluated. This leads to two conclusions: (1) Lower concentrated Ca/Alg results in less stable hydrogels using the LR06 and (2) with each concentration another potential application comes along, some tissues (e.g., vascular tissues) demand stiffer environments than others (e.g., muscle cells). We obtained the best printing results using 3% and 4% Na/Alg. These concentrations are commonly used and described in the literature [11,23,25,43,44,45,46]. Other groups use composites in order to yield stable products [10,23] or complex protocols (e.g., cryogenic 3D printing [17] or FRESH [32]). With our approach, we were able to produce stable Na/Alg structures presenting a feasible method. In addition to that, we could use medium to low concentrated CaCl_2_ solution, as compared to other studies [19,27,33].

The objective of this work was to demonstrate the application of this innovative printing set-up to the materials of different viscosities. In further studies, direct comparison of the printed structures of the same architecture and volume but different viscosities must be performed. Furthermore, bioprinting with two different cell types (hAD-MSCs spheroids and NIH-3T3-GFP) could be accomplished using the LR06. Cells stayed viable for the cultivation period of at least 24 h and the printed constructs neither lost their shape nor dissolved in the media. This proof of principle shows that the LR06 works. In order to characterize the device and the 3D bioprinted products thoroughly, further studies need to be performed. The direct comparison of the printed structures of the same architecture and volume but different viscosities, the shape fidelity (mechanical characterization) over time, as well as the warping and adhesion behavior, need to be evaluated. In addition to that, more sophisticated alginate based hydrogel compositions could be tested with the LR06. For example, the use of cell culture medium instead of deionized water for Na/Alg preparation, which could enhance cell growth and viability [25].

Altogether, the LR06 has proven to be a functional device for sodium alginate bioprinting and it could be used by other researchers working with alginate-based bioinks. In addition, CaCl_2_ mist is suitable to induce polymerization without a negative impact on the cell containing bioink.

## 4. Materials and Methods

### 4.1. Cell Culture

Human adipose tissue-derived mesenchymal stem cells (hAD-MSCs) were isolated from the adipose tissues of patients after abdominoplastic surgery. Informed consent was obtained from all donors and the process was approved by the ethics committee of Hannover Medical School. hAD-MSCs were maintained in alpha-MEM (ThermoFisher Corporation, Waltham, MA, USA) supplemented with 10% human serum (HS) (CC Pro GmbH, Oberdorla, Germany) and 0.5% Gentamycin (Biochrom GmbH, Berlin, Germany). All the cells were incubated at 37 ± 1 °C in a humidified atmosphere of 5% ± 1% (*v*/*v*) CO_2_ in air. The cell number in the trypsinized cell suspension was determined using a haemocytometer.

NIH-3T3 mouse fibroblast cells transduced with a GFP were kindly provided by Prof. Andrea Hoffmann (Hannover Medical School). NIH-3T3 were cultivated in Dulbecco’s Modified Eagle’s Medium (DMEM) (D7777 Sigma-Aldrich, Steinheim, Germany) supplemented with 10% fetal calf serum (FCS) and 100 µg/mL antibiotics (penicillin-streptomycin) in a humidified environment at 37 °C and 5% CO_2_. Only cells of up to ten passages were used in all experiments.

#### hAD-MSCs Spheroids

hAD-MSC-spheroids were created using CELLSTAR^®^ Cell-Repellent Surface cultivation flasks (Greiner Bio-One, Frickenhausen, Deutschland). Cell suspension, eight million cells in 10 mL alpha-MEM (ThermoFisher Corporation, Waltham, MA, USA) supplemented with 10% human serum (HS) (CC Pro GmbH, Oberdorla, Germany) and 0.5% Gentamycin (Biochrom GmbH, Berlin, Germany) was placed into T75 flask for 24 h and the resulting spheroids were centrifuged at 300 g and resuspended in alginates.

### 4.2. Cell Staining (Live/Dead Assay)

For live/dead staining, bioprinted constructs were incubated for 15 min at 37 °C in cell culture medium containing 15 µL Calcein-AM (SigmaAldrich Chemie GmbH, München, Deutschland) and 3 µL propidium iodide (SigmaAldrick Corporation, St. Louis, MO, USA) per 1.482 mL alpha-MEM medium. Micrsocopic images were taken with fluorenscence microscope Olympus IX 50 (Olympus Corporation, Tokyo, Japan) with the camera (Olympus SC30, IX-TVAD, Tokyo, Japan) and CellSens Software (CellSens Standard 1.7.1, Olympus).

### 4.3. Cell Toxicity Evaluation of Nebulized Media and CellTiterBlue^@^ Assay

Cell toxicity experiment evaluation is based on the ISO 10993-12:2012 (Biological evaluation of medical devices—Part 12: Sample preparation and reference materials) protocoll. Here, a reduction of cell viability after treatment with extraction media of >20% indicates a toxic effect on the cells. The operation procedure for the ISO protocol-derived assay is as follows: 20 µL media were nebulized in the respective vessel for 1 min and was used for the cultivation of AD-MSCs (100 µL and 8000 cells per well) in 96 well plates for 24 h at 37 ± 1 °C in a humidified atmosphere of 5% ± 1% (*v*/*v*) CO_2_ in air. After medium replacement with CTB^®^ solution cell culture medium (without nebulization) was incubated for 72 under the same conditions served as control.

CellTiter-Blue^®^ Cell Viability Assay (Promega Corporation, Fitchberg, WI, USA) was conducted according to manufacturer instructions: Briefly, working solution of CellTiter-Blue^®^-reagent in DMEM/alpha-MEM without antibiotics and serum was prepared: 10% CellTiterBlue^®^-reagent in DMEM or alpha-MEM. 100 µL of CTB^®^-working solution was added into each well. Additionally, two wells without cells were filled with CellTiter-Blue^®^ working solution for the blank measurement. Plates were incubated at 37 ± 1 °C in a humidified atmosphere of 5% ± 1% (*v*/*v*) CO_2_ in air for 105 min. Fluorescence was measured with a microplate fluorometer (Ex579 nm/Em584 nm) (Fluoroskan Ascent, Thermo Electron Corporation, Waltham, MA, USA).

### 4.4. CAD Modeling and 3D Printing Techniques

#### 4.4.1. Nebulizer

The nebulizer used in this study is a custom design. It is assembled of two parts: (1) 3D printed bioprinter-mount with mist chamber and (2) the nebulizer. The latter is a waterproof ultrasonic mist generator (Sens humidity 12 V nebulizer mod, NB80E-01-H, TDK Corporation, Tokyo, Japan). The settings of the piezo element were set once and used for all presented results: 12 V operating voltage, 40 mm CaCl_2_ solution level, 1600 Hz. Please see Figure 1 for further details.

For design, all 3D printed parts were constructed with computer-aided-design (CAD) software Autodesk Inventor Professional 2015 (Autodesk Inc., San Rafael, CA, USA). The nebulizer is designed to be mounted in an Allevi Biobot 1 bioprinter. As rapid prototyping for preliminary experiments (fitting to Biobot 1 bioprinter, CaCl_2_ mist tests), the fused deposition modeling (FDM) 3D printing technique was used. Therefore, the nebulizer was manufactured with a Makerbot Z18 (Stratasys, Eden Prairie, MN, USA), as printing material poly lactic acid (PLA) was deployed (printing time 14 h at 0.2 mm layer height). The final product, which was used for bioprinting experiments, was 3D printed using a high definition multi jet 3D printer (MJP) ProJet 2500 (3D Systems, Rock Hill, SC, USA) and sliced with 3D Sprint software (3D Systems; Rock Hill, SC, USA). Printing material was VisiJet M2-RCL [4] (printing time 21 h at 30 µm layer height). The used 3D printing material was tested both on its chemical stability and biocompatibility in previous studies and published by Raddatz et al. in 2017 [4].

#### 4.4.2. Bioink Preperation

As bioink 0.5%, 1%, 2%, 3% and 4% (*w*/*v*) sodium alginate solution (0.5; 1; 2, 3, and 4 g CaCl_2_ in 100 mL deionized water) was used and heat steam sterilised before further application. After cultivation as described above, the cells (hAD-MSCs, NIH-3T3-GFP) were centrifuged (5 min, 300× *g*) and resuspended in the respective hydrogel concentration (5 × 10^6^ cells/mL). Before centrifugation, NIH-3T3-GFP were detached from the cell culture surface using 4 mL Trypsin solution. Finally, the bioink was transferred to 10 mL syringes and instantly mounted in the bioprinter and used for bioprinting.

#### 4.4.3. 3D Bioprinting

3D bioprinted constructs were designed using CAD software mentioned in Section 4.4 and sliced with Repetier Host. For 3D bioprinting, the robotic dispensing bioprinter Biobot 1 (Allevi Inc., Philadelphia, PA, USA) was used.

Bioprinting took plase in petri dishes (Greiner Bio-One International GmbH, Kremsmünster, Österreich). Therefore, the nebulizer was mounted on the bioprinter’s building platform, as seen in Figure 1B. In order to improve surface hydrophilicity and gain better printing results, the surface was roughened using abrasive paper (3M Lapping Film Bogen x261, 3 µm grain size, 3M Deutschland GmbH, Neuss, Germany). Different printing nozzles were tested and 0.256 mm diameter nozzles (Nordson Corporation, Westlake, OH, USA) turned out to yield the best results (not shown). Gelation was induced by nebulized 500 mM CaCl_2_. Further printing parameters are listed in Table 1.

Before bioprinting all devices and material were sterilized. The bioprinter, petri dishes, and the 3D printed nebulizer were sterilized prior to the experiment (rinsed with 70% (*v*/*v*) isopropanol and 30 min under UV radiation). CaCl_2_ solution was sterilized using sterile filtration. Assembly of the nebulizer and actual bioprinting were conducted in sterile environments in a laminar flow cabinet.

Bioprinting work flow: After assembly of the nebulizer and its mounting in the bioprinter, the syringe is filled with hydrogel (containing the hAD-MSC spheroids and NIH-3T3-GFP, respectively). After the 3D print has ended, the construct is exposed to the CaCl_2_ mist for 30 s. Afterwards, the petri dish with the bioprinted construct is washed with PBS twice and 250 µL cell culture media (alpha-MEM) and then incubated at 37 ± 1 °C in a humidified atmosphere of 5% ± 1% (*v*/*v*) CO_2_ in air.

### 4.5. Rheology

Alginate solutions for rheology experiments were prepared as described in Section 4.4.2. Measurements were performed with a MCR302 (Anton Paar GmbH, Graz, Austria) rheometer equipped with plate-plate geometry and a temperature-controlled peltier element. Each concentration was measured in three replicates.

#### 4.5.1. Viscosity

Viscosity measurements were performed with rotational viscometry with a measuring plate of 40 mm diameter and a gap size of 1 mm. The sample (1.4 mL) was pipetted onto the measuring plate and was allowed to equilibrate for 2 min at 37 °C. Measurements were performed at 37 °C with a logarithmic increase of the shear rate [1/s] at 41 time points 0–1000 1/s.

#### 4.5.2. Storage Modulus

The storage modulus was determined for model constructs of Ca/Alg discs that were fabricated in the dimensions matching the plate geometry of the rheological instrument. For this purpose, Na/Alg solution of appropriate concentration was poured in silicone casting molds (2 cm diameter, 1 mm height). The constructs could not be crosslinked by the nebulizer as the height of 1 mm provided too high of a diffusion barrier and the solution remained liquid. Therefore, direct addition of CaCl_2_ solution had to be performed.

Storage modulus measurements were performed wih a time sweep in an oscillation experiment with constant 0.3% (amplitude gamma) and a frequency of 1 Hz in the linear viscoelastic range of the alginate hydrogel. Measurements were performed at 37 °C with a measuring plate of 20 mm, a gap size of 1 mm, and a constant load of 1 N. Values shown are averages of six individual measurements.

### 4.6. Chemicals

Used bulk chemicals were purchased from Sigma-Aldrich Corp. (Taufkirchen, Germany). Deionized water was prepared with Arium^®^ (Sartorius Stedim Biotech GmbH, Göttingen, Germany). Sodium alginate (A2033, 100 kDa, M/G ratio: 1.56) was purchased at Merck KGaA (Darmstadt, Germany).

### 4.7. Statistical Analysis

For the statistical analysis, ANOVA (One Way, Bonferroni) was used. The confidence interval is 99%. As operating software Origin 2018 Version 95G (Origin LabCorporation, Northampton, MA, USA) was used. All data represented as mean ± SD for measurements for each sample.

## Figures and Tables

**Table 1:** Bioprinting parameters for different Na/Alg hydrogel concentrations.

Na/Alg [%, (*w*/*v*)]	Printing Temperature	Printing Platform Temperature	Air Pressure for Extrusion		CaCl_2_ [c]
			[PSI]	[mPa]	
0.5	37 °C	RT (up to 40 °C possible)	N/A	N/A	500 mM
1			<1.5	<10.3	
2			1.8–2.4	12.4–16.5	
3			2.1–2.8	14.5–19.3	
4			9.9–13.1	68.2–90.3	

## Supplementary Materials

The following are available online at http://www.mdpi.com/2079-4983/9/4/63/s1, File S1: LR06-Chamber, File S2: LR06-Lid, Video S1: Demonstration-Video, Figure S1: Object-processing.
