# Supplementary figures and images for: Development and Application of an Additively Manufactured Calcium Chloride Nebulizer for Alginate 3D-Bioprinting Purposes
Source: J Funct Biomater. 2018 Nov 9;9(4):63. doi: 10.3390/jfb9040063 (PMC6306849; doi:10.3390/jfb9040063)

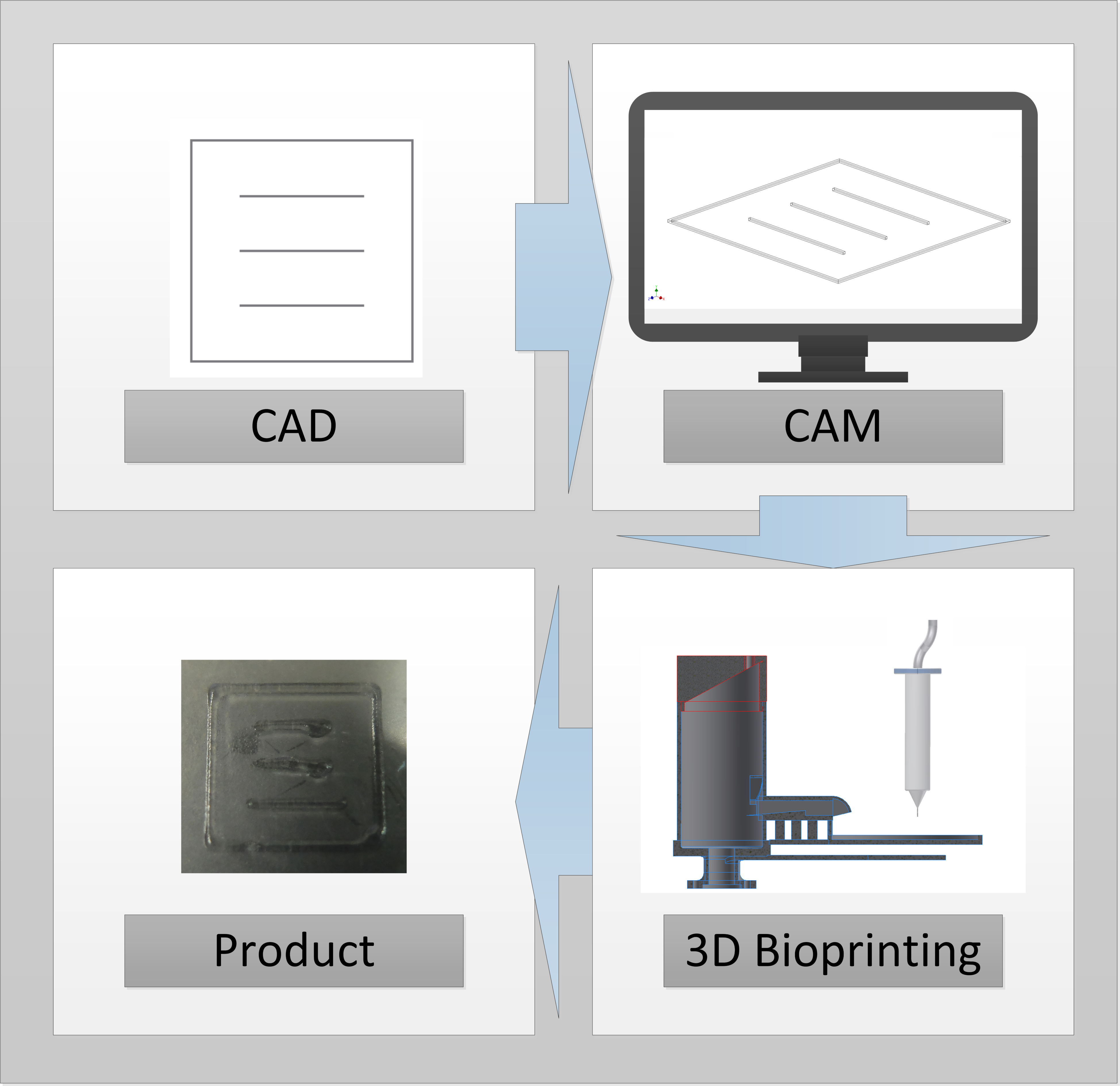

Supplement: Supplementary file 1 [file jfb-09-00063-s001.zip › jfb-354541-supplementary/Figure S1 Object-manufacturing.tif]
